# Allergic diseases of the skin and drug allergies – 2002. Association of food and drug allergy with anti-tuberculosis drug related hepatitis or skin reactions

**DOI:** 10.1186/1939-4551-6-S1-P92

**Published:** 2013-04-23

**Authors:** Anoma Siribaddana, Asanga Upul, Kokila Kodithuwakku, Gayani Pathirana, Dinesh Dassanayaka

**Affiliations:** 1 Respiratory Medicine, Teaching Hospital Kandy, Kandy, Sri Lanka

## Background

This study was carried out to find out whether allergy to drug or food acts as a risk factor for the development of anti-tuberculosis drug (ATT) induced hepatitis or skin reactions.

## Methods

This case control study was conducted in the Respiratory Unit, Teaching Hospital Kandy Sri Lanka Around 800 new tuberculosis patients are diagnosed in this center annually. The study was carried out for a period of one year from 1^st^ July 2010 to 30^th^ June 2011.

The patients with tuberculosis who presented to the Respiratory clinic due to anti-tuberculosis drug induced hepatitis or skin reactions while on INAH, rifampicin, ethambutol and pyrazinamide (Category 1 regime) and not on long term steroids and anti-histamines were studied . Patients with decompensated cirrhosis and active liver disease prior to start of ATT, patients with active skin diseases prior to treatment and Patients with HIV infection were excluded.

ATT induced hepatitis was diagnosed by the definition used by Rohit Singla etal. [1]

ATT induced skin reactions were defined as presence of itching, rash or skin eruption that occurred after commencing ATT and disappearing following withdrawal of ATT.

Controls were patients with tuberculosis on category 1 regime, and who didn’t develop hepatitis or skin reactions during the 6 month treatment period.

Cases and controls were inquired for the presence of allergy to drugs or food and were recorded using an interviewer administered questionnaire. Controls were matched with cases for age, gender, weight and consumption of alcohol. Cases and controls were compared using odds ratio with confidence intervals.

## Results

61 cases and 61 controls were studied. Average age of cases was 47.4 (SD 15.1). There were 66 males [54.1%] (and 56 [45.9%] females. Out of 61 cases 41 (67.3%) had ATT induced hepatitis and 20 (32.7%) had skin reactions. The odds ratio was 5.8%.

## Conclusions

Patients with allergy to drug or food have 5.8 times risk of developing anti-tuberculosis drug induced hepatitis or skin reaction during standard regime for tuberculosis.
